# Anaesthetic management using remimazolam in a patient with severe aortic stenosis: a case report

**DOI:** 10.1186/s12871-021-01422-6

**Published:** 2021-08-14

**Authors:** Minako Furuta, Hisakatsu Ito, Mitsuaki Yamazaki

**Affiliations:** Department of Anaesthesiology, Toyama University Hospital, 2630 Sugitani, Toyama, 930-0194 Japan

**Keywords:** remimazolam, aortic stenosis, circulatory dynamics

## Abstract

**Background:**

The administration of general anaesthesia in patients with aortic stenosis (AS) requires careful attention to haemodynamics. We used remimazolam for the induction and maintenance of anaesthesia in a woman with severe AS undergoing a total mastectomy.

**Case presentation:**

An 81-year-old woman with severe AS was scheduled to undergo a total mastectomy. We decided to administer total intravenous anaesthesia with remimazolam to minimize haemodynamic changes. Although the patient showed transient hypotension after anaesthesia induction, the cardiac index was preserved with a low dose of continuous noradrenaline. The anaesthesia was then safely maintained without a decrease in the patient’s cardiac index.

**Conclusions:**

General anaesthesia using remimazolam preserved cardiac output in this patient; therefore, remimazolam can be safely used to avoid the risk of cardiac suppression in patients with severe AS.

## Background

The administration of general anaesthesia in patients with aortic stenosis (AS) requires careful attention to haemodynamics. In AS, circulation is maintained by high left ventricle pressure from increased preload and contractile force against high outflow resistance. General anaesthetic agents, often lead to fatal circulatory failure in AS, because they suppress cardiac contractility and reduce preload due to vasodilation, yet do not alter the high afterload created by the reduction in the valve area [1]. The degrees of vasodilatation and suppression of cardiac contraction by benzodiazepines are limited [2]; however, its duration of action has been demonstrated to increase with age, obesity, and renal and hepatic insufficiency [3, 4]. Remimazolam is an ultrashort-acting benzodiazepine that is newly approved for the induction and maintenance of general anaesthesia in Japan, which is also expected to cause fewer haemodynamic changes [5]. However, previous clinical reports on anaesthetic management using remimazolam in patients with AS are lacking. In this case, we used remimazolam for the induction and maintenance of anaesthesia for a woman with severe AS undergoing a total mastectomy.

## Case presentation

An 81-year-old woman (height, 145 cm; weight, 65 kg) with severe AS was scheduled to undergo a total mastectomy with a sentinel lymph node biopsy. Preoperative transthoracic echocardiogram showed a maximal blood flow velocity of 4.4 m/s. The calculated aortic valve area and mean pressure gradient were 0.76 cm^2^ and 43 mmHg, respectively. Left ventricular systolic function was preserved (ejection fraction, 68%). The patient had undergone percutaneous coronary intervention for effort angina 3 months prior to presentation. In addition, she had a history of hypertension, diabetes, and hyperlipidaemia. Her preoperative laboratory test indicated a severe decline in the estimated glomerular filtration rate (21.4 mL/min/1.73 m^2^) and haemoglobin level (7.4 g/dL).

**Fig. 1:**
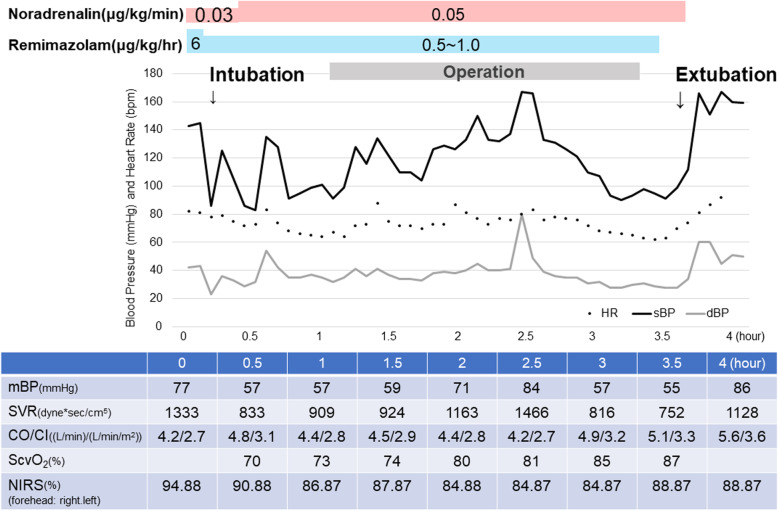
Anaesthesia record. Change in haemodynamics during general anaesthesia. HR: heat rate; sBP: systolic blood pressure; dBP: diastolic blood pressure; mBP: mean blood pressure; SVR: systemic vascular resistance; ScvO2: central venous oxygen saturation; CO: cardiac output; CI: cardiac index; NIRS: near-infrared spectroscopy

Continuous arterial pressure was measured after inserting a catheter into the radial artery, and haemodynamic monitoring was performed using arterial pressure-based cardiac output (FloTrac; Edwards Life Sciences, LLC, Irvine, CA, USA). After establishing the bispectral index (BIS) and conducting near-infrared spectroscopy in addition to standard monitoring, general anaesthesia was induced by intravenous remimazolam (6 mg/kg/h) and remifentanil (0.3 μg/kg/min) along with continuous administration of noradrenaline (0.03 μg/kg/min) to avoid undesirable vasodilation. The patient lost consciousness 171 s after drug administration, and we reduced the dose of remimazolam to 1 mg/kg/h. Rocuronium (50 mg) was administered followed by tracheal intubation. An oximetry catheter (PreSep; Edwards Life Sciences, LLC, Irvine, CA, USA) was inserted via the right jugular vein to measure central venous oxygen saturation. Anaesthesia was maintained with remimazolam adjusted from 0.5 to 1.0 mg/kg/h to maintain the BIS between 40 and 60. Haemodynamic changes during surgery are shown in Fig. 1. The patient showed transient hypotension after anaesthesia induction. Mean blood pressures, cardiac output, index, and systemic vascular resistance were 77 mmHg, 4.2 L/min, 2.7 L/min/m^2^, and 1,333 dyne*sec/cm^5^ before administrating remimazolam, and 57mmHg, 4.8 L/min, 3.1 L/min/m^2^, and 833 dyne*sec/cm^5^ after administration. Systemic vascular resistance was calculated as assuming central venous pressure was 7 mmHg. Remimazolam administration continued until the end of the surgery. The patient regained consciousness 9 min later, was extubated 18 min later, and restored orientation 28 min later. The duration of anaesthesia was 229 min, and the operation time was 142 min. The patient was monitored in the intensive care unit (ICU) overnight, and she was discharged from the ICU the next day.

## Discussion and conclusions

Haemodynamic stability is one of the advantages of remimazolam [5]. Total intravenous anaesthesia with remimazolam results in increased systolic and diastolic blood pressures and a reduced percentage of patients using vasopressors than anaesthesia with propofol [6]. Although remimazolam reduced blood pressure in a dose-dependent manner, it did not reduce the cardiac index in a pre-clinical study in sheep [7]. Therefore, the reduction in blood pressure observed with remimazolam can be attributed to reduced peripheral vascular resistance. Although the induction dose of remimazolam is generally 12 mg/kg/h, lower doses are recommended for frail elderly or ASA class 3 or higher patients [8]. Even 6 mg/kg/h remimazolam was successful at inducing anaesthesia in all critically ill patients in ASA class III or higher [9]. One study reported that general anaesthesia for cardiac surgery was induced with 6 mg/kg/h remimazolam [10]. We selected 6 mg/kg/h as the induction dose based on the patient’s cardiac condition. Further study is needed to determine the suitable induction dose of remimazolam for cardiac and non-cardiac surgeries.

In the current case, we administered a continuous injection of noradrenaline starting at the induction of anaesthesia to prevent a reduction in vascular resistance. Although the patient showed transient hypotension after anaesthesia induction, the cardiac index was preserved without the administration of additional inotropic agents. During maintenance of anaesthesia, the cardiac index never decreased, and regional cerebral oxygen saturation on near-infrared spectroscopy was preserved. The central venous oxygen saturation level increased, which could have been influenced by an increased fraction of inspiratory oxygen or the transfusion of red blood cells. During anaesthesia in patients with severe heart disease, such as this patient, rapid haemodynamic changes can cause critical circulatory disruptions. Thus, an anaesthetic agent with low circulation-suppressing effects, such as remimazolam, should be selected for such patients, accompanied by intensive monitoring and inotrope therapy.

Propofol and barbiturates, which are common agents used to induce anaesthesias, often lead to fatal circulatory failure in AS patients because they directly activate the chloride channel of the GABA_A_ receptor in the absence of GABA, rapidly suppress cardiac contractility and reduce pre-load due to vasodilation. In contrast, benzodiazepines cause limited vasodilatation and cardiac depression [2]. In terms of the mechanism, the reaction is provoked by a mild allosteric reaction that increases the affinity of GABA to the receptor [11].

Etomidate is also considered to be a relatively safer sedative agent for patients with cardiac disease [12]. In a previous study, etomidate was twice as less likely as propofol to cause hypotension after anaesthesia induction in patients with severe AS; however, etomidate decreased the cardiac index to the same extent as propofol [1]. Although the context-sensitive half-life of the continuous infusion of etomidate is shorter than that of propofol, prolonged etomidate infusion tends to be contraindicated because of adrenal toxicity [13]. Furthermore, etomidate should be carefully administered due to delayed emergence in patients with hepatic or renal dysfunction because it is metabolized in the liver and excreted by the kidney [14]. However, remimazolam undergoes organ-independent metabolism by tissue esterases to form an inactive metabolite [3]. In addition, remimazolam has the advantage of being antagonized by flumazenil.

Ketamine is known to cause increases in blood pressure and the pulse rate because it increases the plasma level of noradrenaline, which may make it suitable for the induction of anaesthesia in patients with AS [15]. However, it can cause hallucinations and nightmares, especially in elderly individuals [16]. Furthermore, we were concerned that ketamine-induced tachycardia would exacerbate the myocardial ischaemia in the patient in the present case. It should be used with caution in patients with coronary heart disease.

It should be noted that a clear tapering regimen has not been established for remimazolam, unlike propofol [3]. In this case, we adjusted the dose of remimazolam to maintain the BIS levels between 40 and 60 based on the results from a clinical trial [6]. In a clinical phase I trial targeting healthy Japanese volunteers, 95% of the subjects lost consciousness upon remimazolam infusion with a BIS value of 53, indicating that the BIS could be useful for the assessment of consciousness [9]. However, benzodiazepines are known to lead to slightly higher BIS levels than propofol [17]. Therefore, it has been noted that higher BIS values might be sufficient for general anaesthesia with remimazolam than propofol. However, as mentioned above, remimazolam has an advantage over other intravenous anaesthetics in that its effects can be antagonized by flumazenil [3].

The rapid onset/offset of remimazolam can be potentially helpful for fast-tracking cardiac surgery patients. As mentioned above, attention should be given to vasodilation at induction. Delayed awakening of more than 30 minutes has been reported to occur in approximately 8% of patients after the end of remimazolam administration [6]. Although flumazenil can antagonise remimazolam in such cases, there may be a rapid increase in vascular resistance due to the rapid offset, and caution should be exercised in patients with cardiac complications.

The most common adverse effects of remimazolam are hypotension, followed by bradycardia. In addition, headache and nausea can occur as side effects. Although the frequency is unknown, there is a possibility that dependence may develop, and attention should be given to withdrawal symptoms after the end of administration.

As a limitation of this case report, continuous noradrenaline administration was started at the same time as the administration of remimazolam, so the degree of vasodilatation due to remimazolam could not be accurately assessed.

Based on our experience of this case, general anaesthesia using remimazolam may preserve cardiac output in patients with severe AS when used with a norepinephrine infusion. Remimazolam can be useful for the general anaesthesia management of patients with severe AS if attention is given to vasodilation. However, remimazolam still has other limitations such as passible side effects of headache, nausea and dependence, unclear tapering regimen and delayed awakening without flumazenil, besides vasodilation. Further clinical research on this topic is warranted.

## Data Availability

Not applicable

## Abbreviations

AS: Aortic stenosis
BIS: Bispectral index
ICU: Intensive care unit
